# Get to the heart of pediatric kidney transplant recipients: Evaluation of left- and right ventricular mechanics by three-dimensional echocardiography

**DOI:** 10.3389/fcvm.2023.1094765

**Published:** 2023-03-17

**Authors:** Zsuzsanna Ladányi, Adrienn Bárczi, Alexandra Fábián, Adrienn Ujvári, Orsolya Cseprekál, Éva Kis, György Sándor Reusz, Attila Kovács, Béla Merkely, Bálint Károly Lakatos

**Affiliations:** ^1^ Heart and Vascular Center, Semmelweis University, Budapest, Hungary; ^2^ 1st Department of Pediatrics, Semmelweis University, Budapest, Hungary; ^3^ Department of Surgery, Transplantation and Gastroenterology, Semmelweis University, Budapest, Hungary; ^4^ Department of Pediatric Cardiology, Gottsegen György Hungarian Institute of Cardiology, Budapest, Hungary

**Keywords:** echocardiography, kidney transplantation, pediatric patients, echocardiography - 3 dimensional, speckle-tracking analysis

## Abstract

**Background:**

Kidney transplantation (KTX) markedly improves prognosis in pediatric patients with end-stage kidney failure. Still, these patients have an increased risk of developing cardiovascular disease due to multiple risk factors. Three-dimensional (3D) echocardiography allows detailed assessment of the heart and may unveil distinct functional and morphological changes in this patient population that would be undetectable by conventional methods. Accordingly, our aim was to examine left- (LV) and right ventricular (RV) morphology and mechanics in pediatric KTX patients using 3D echocardiography.

**Materials and methods:**

Pediatric KTX recipients (*n* = 74) with median age 20 (14–26) years at study enrollment (43% female), were compared to 74 age and gender-matched controls. Detailed patient history was obtained. After conventional echocardiographic protocol, 3D loops were acquired and measured using commercially available software and the ReVISION Method. We measured LV and RV end-diastolic volumes indexed to body surface area (EDVi), ejection fraction (EF), and 3D LV and RV global longitudinal (GLS) and circumferential strains (GCS).

**Results:**

Both LVEDVi (67 ± 17 vs. 61 ± 9 ml/m^2^; *p* < 0.01) and RVEDVi (68 ± 18 vs. 61 ± 11 ml/m^2^; *p* < 0.01) were significantly higher in KTX patients. LVEF was comparable between the two groups (60 ± 6 vs. 61 ± 4%; *p* = NS), however, LVGLS was significantly lower (−20.5 ± 3.0 vs. −22.0 ± 1.7%; *p* < 0.001), while LVGCS did not differ (−29.7 ± 4.3 vs. −28.6 ± 10.0%; *p* = NS). RVEF (59 ± 6 vs. 61 ± 4%; *p* < 0.05) and RVGLS (−22.8 ± 3.7 vs. −24.1 ± 3.3%; *p* < 0.05) were significantly lower, however, RVGCS was comparable between the two groups (−23.7 ± 4.5 vs. −24.8 ± 4.4%; *p* = NS). In patients requiring dialysis prior to KTX (*n* = 64, 86%) RVGCS showed correlation with the length of dialysis (*r* = 0.32, *p* < 0.05).

**Conclusion:**

Pediatric KTX patients demonstrate changes in both LV and RV morphology and mechanics. Moreover, the length of dialysis correlated with the contraction pattern of the right ventricle.

## 1. Introduction

For children with end-stage kidney disease (ESKD), kidney transplantation (KTX) is the best treatment option in terms of long-term outcomes and the quality of life. Survival statistics among pediatric transplant recipients have had a significant improvement in recent decades due to the advancements in surgical care, the progress in post-KTX immunosuppressive management and infection control, and the improvement in cardiovascular care (1, 2).

Following infections as the most frequent etiology, cardiovascular disease (CVD) is the second most common cause of death for pediatric ESKD patients (3). Albeit KTX is effective in reducing the morbidity of CVD, transplant recipients are still at a significantly higher risk of dying from CVD compared to the age-matched controls (4, 5). Beyond pre-transplant ESKD-related injury of the cardiovascular system, these individuals are also exposed to various KTX-related factors, such as the cardiovascular effects of immunosuppressive agents and progressively deteriorating graft function, along with “classical” risk factors such as hypertension and increased arterial stiffness (6–9).

Hence, to prevent later cardiovascular events, the early detection of cardiovascular dysfunction is essential (7). Often minor, but detectable changes in the cardiac structure and function already manifest in children with chronic kidney disease (CKD) who still only have mild or no symptoms (10).

Novel imaging techniques, such as left ventricular (LV) speckle-tracking echocardiography-derived global longitudinal (GLS) and circumferential strain (GCS) have the ability to detect subclinical functional deterioration, even when conventional functional parameters, such as ejection fraction (EF), are still preserved (11, 12). Speckle-tracking deformation imaging also provides incremental information regarding right ventricular (RV) function in a wide variety of patients, including patients with kidney disease (13). Consequently, speckle-tracking echocardiography is highly recommended by current guidelines and strain measurements are increasingly implemented in the everyday clinical routine.

Three-dimensional (3D) echocardiography allows an even more detailed assessment of the heart and may unveil even subtle functional and morphological changes of the chambers. This technique provides a more precise quantification of ventricular volumes and EF while being highly reproducible and shows good agreement with the gold-standard cardiac magnetic resonance imaging-derived values (14). Moreover, it also offers accurate strain measurement methods to describe the contractile function of the heart, more recently not just in the left ventricle (15, 16), but also in its right counterpart (17, 18).

Accordingly, our aim was to examine LV- and RV morphology and mechanics in childhood kidney transplantation patients using 3D echocardiography.

## 2. Materials and methods

### 2.1. Study population

Initially, 86 KTX patients transplanted in childhood were enrolled between December 2016 and October 2018 in this cross-sectional study. The post-transplant care of the patients is performed by the 1st Department of Pediatrics of Semmelweis University, prospectively referring them to the Heart and Vascular Center of Semmelweis University for an echocardiographic examination. During this visit blood samples were also taken and detailed medical history and demographic data were obtained. We reviewed the etiology of the KTX patients’ primary kidney disease, their history of dialysis, graft source and medication from electronic medical records. Glomerular filtration rate (eGFR) was calculated according to the Schwartz formula or the CKiD U25 formula when analysing patients up to 25 years of age.

Inclusion criteria consisted of having a functioning allograft (no need for dialysis), and no history of cardiac disease. Exclusion criteria were poor echocardiography image quality (*n* = 9) and an established diagnosis of significant cardiac disease (hypertrophic cardiomyopathy; *n* = 1, hemodynamically significant aortic regurgitation; *n* = 2). The final study population consisted of 74 KTX patients [age: 20 (14–26) years, under 18 years: *n* = 40 patients] transplanted in childhood (age at KTX: 10 ± 4 years). An age- and gender-matched healthy population served as controls (CTR) [*n* =74, age 20 (13–23) years].

### 2.2. Two-dimensional echocardiography

Conventional echocardiographic exams were performed using a GE Vivid E95 (equipped with a 4V-D matrix-array transducer; GE Healthcare, Horten, Norway) or a Phillips Epiq 7G (equipped with an X5-1 matrix-array transducer; Phillips Medical Systems, Best, Netherlands) ultrasound system. A standard acquisition protocol consisting of loops from parasternal, apical, and subxiphoid views was used according to current guidelines (19). LV wall thicknesses and diameters were evaluated in parasternal long-axis view. Relative wall thickness (RWT) was calculated as end-diastolic posterior wall thickness multiplied by 2 and divided by LV end-diastolic internal diameter. Pulsed wave Doppler interrogation at the level of the mitral valve coaptation was obtained to determine early (E) and late diastolic (A) peak LV inflow velocities and their ratio (E/A) and deceleration time (DT). Systolic (s’), early (e′), and late diastolic (a′) velocities at the mitral lateral and medial annuli and the tricuspid free wall annulus were measured using pulsed-wave tissue Doppler imaging. LV filling pressure was estimated by dividing the transmitral E wave with the tissue Doppler imaging-derived averaged lateral and medial annular e’. RV basal diameter was measured as the maximal transverse dimension in the basal third of the RV inflow from the RV-focused apical four-chamber view in the end-diastolic frame. M-mode-derived tricuspid annular plane systolic excursion (TAPSE) was calculated as the maximum longitudinal displacement of the tricuspid annulus. Atrial volumes were estimated using the Simpson method. The left atrial volume index (LAVi) and right atrial volume index (RAVi) were normalized to the body surface area (BSA). Peak pulmonary artery systolic pressure (PASP) was calculated from the tricuspid regurgitant jet signal velocity and right atrial pressure, which was estimated using inferior vena cava diameter and collapsibility (REF).

### 2.3. Three-dimensional echocardiography

Beyond the routine echocardiographic protocol, ECG-gated full-volume 3D data sets reconstructed from four or six cardiac cycles optimized for the right or left ventricle were obtained for offline analysis. Image quality was verified at the bedside to avoid “stitching” and “dropout” artefacts of the 3D data. Further measurements were performed on a separate workstation using dedicated software (4D RV-Function 2 and 4D LV-Analysis 3; TomTec Imaging, Unterschleissheim, Germany). The software detects the endocardial surface of the left- and right ventricles, and following manual correction, it traces its motion throughout the cardiac cycle. We determined the end-diastolic volume index (EDVi), end-systolic volume index (ESVi), and stroke volume index (SVi) normalized to BSA, and to characterize global LV and RV functions, EFs were also assessed. By tracing the end-diastolic epicardial contour we measured LV mass index (LVMi) normalized to BSA. The dedicated 3D LV analysis software also enables 3D speckle-tracking analysis, therefore, LVGLS and LVGCS were also calculated. The software automatically measures LV twist and torsion using basal and apical short axis deformation, and systolic dyssynchrony index (SDI) as the standard deviation of the time takes to reach minimum systolic volume for each LV segment.

### 2.4. The ReVISION method

Our custom 3D RV deformation analysis was previously described in detail (17, 18). All analyses were performed on a standard computer. The input to our software (ReVISION method; Argus Cognitive, Lebanon, New Hampshire) is a series of 3D meshes exported from TomTec 4D RV-Function 2, taken at even time instants during the cardiac cycle.

The analysis begins with the reorientation of the 3D RV mesh. A local coordinate system is defined for each mesh series, where the basis vectors correspond to the longitudinal, radial and anteroposterior directions.

Second, the wall motions of the 3D RV model are decomposed in a vertex-based manner (e.g., for quantifying the magnitude of longitudinal motion, only the movement of the vertices along the vertical axis is taken into account). This decomposition step transforms the original mesh series into several series of meshes, each corresponding to a decomposition type (i.e., which movement directions are enabled and which ones are not).

In the final step, by the decomposition of the model's motion along the three anatomically relevant axes, we can measure the volume change of the right ventricle attributable to the specific direction separately. To assess GCS, we create 15 equidistant circumferential contours on the first mesh of each series and calculate their positions at later time instants. For GLS, 45 longitudes are generated by connecting the apex, the predefined vertices of the base, and vertices in equidistant latitude to the latter. The pulmonary and tricuspid annular planes are excluded from this part of the analysis. Our software calculates 3D RV GCS and GLS by averaging the length changes of each circumferential contour or longitude referenced to their end-diastolic length. Global area strain (GAS) is also calculated by the relative change of the endocardial surface between end-diastole and end-systole. The software also allows the measurement of segmental strain values, therefore, longitudinal, circumferential and area strains of the septum and the free wall were also calculated.

### 2.5. Statistical analysis

We performed statistical analysis using STATISTICA version 13.4 (TIBCO Software Inc, Palo Alto, CA, United States). The normal distribution of our variables was verified using the Shapiro–Wilk test. Data are presented as mean ± standard deviation (SD), median (interquartile ranges), or percentage, as appropriate. Groups were compared with the unpaired Student's *t*-test or Mann–Whitney *U* test for continuous variables and the chi-square or Fisher's exact test for categorical variables according to normality. Correlations between variables were evaluated by Pearson's rank correlation test or non-parametric Spearman correlation. *p*-values < 0.05 were considered to be statistically significant.

Intra- and interobserver variability of the most relevant parameters were also assessed. The operator of the first measurements (BL) and a second expert reader (ZL), both blinded to the study groups, repeated the measurements in a randomly chosen subset of 5–5 subjects from each group. We calculated Lin's concordance correlation coefficient and coefficient of variation.

## 3. Results

### 3.1. Patient characteristics

The baseline statistics of the KTX and CTR groups are presented in Table 1.

**Table 1:** Baseline characteristics of the kidney transplant and the control groups.

	Kidney transplant (*n* = 74)	Control (*n* = 74)	*p*-value
Age (years)	20 (14–26)	20 (13–23)	0.769
Female (*n*)	32	34	0.741
Height (cm)	160 (152–172)	168 (159–178)	**0.005**
Weight (kg)	58 ± 19	60 ± 17	0.525
BSA (m^2^)	1.6 ± 0.3	1.7 ± 0.3	0.195
SBP (mmHg)	122 (112–131)	114 (103–125)	**0.016**
DBP (mmHg)	70 (63–80)	66 (60–76)	0.176
HR (1/min)	77 ± 12	76 ± 14	0.533
Pre-transplant dialysis, *n* (%)	64 (86)	-	
Hemodialysis, *n* (%)	26 (35)	-	
Peritoneal dialysis, *n* (%)	50 (68)	-	
Both hemodialysis and peritoneal dialysis, *n* (%)	12 (16)	-	
Length of need for dialysis (months)	9.4 (3.6–18.1)	-	
Age at KTX (years)	10 ± 4	-	
Time since KTX (months)	112 ± 82	-	

Data are presented as mean ± SD, median (interquartile range) or number of patients. Values with a significant difference are presented in bold. BSA, body surface area; SBP, systolic blood pressure; DBP, diastolic blood pressure. HR, heart rate; KTX, kidney transplantation.

There was no significant difference regarding the age, gender, weight, BSA and heart rate of the two groups. However, the controls were significantly taller than the transplant patients. Furthermore, the KTX group presented with significantly higher systolic blood pressure, while the diastolic blood pressure did not differ.

In the KTX group, 86% (*n* = 64) received dialysis prior to transplant, 41% of whom (*n* = 26) was treated with hemodialysis. Preemptive transplant occurred in 14% (*n* = 10) of the patients. Their mean age at KTX was 10 ± 4 years, and 112 ± 82 months passed since the transplantation.

The underlying primary kidney disease were as follows: kidney agenesis or dysplasia (*n* = 14), polycystic kidney disease (*n* = 9), obstructive uropathy (*n* = 4), focal segmental glomerulosclerosis (*n* = 14), nephronophthisis (*n* = 8), chronic glomerulonephritis (*n* = 7), pyelo- or interstitial nephritis (*n* = 5), congenital disorders (*n* = 6), miscellaneous (*n* = 5) and unknown etiology (*n* = 2).

The additional clinical characteristics of the transplant patients are shown in Supplementary Table S1.

### 3.2. 2D conventional echocardiography and tissue Doppler imaging

The conventional 2D echocardiographic parameters of the patients are summarized in Table 2.

**Table 2:** Conventional echocardiographic left- and right heart parameters in the kidney transplant and the control groups.

	Kidney transplant (*n* = 74)	Control (*n* = 74)	*p*-value
LVIDd (mm)	45.0 ± 5.5	45.5 ± 4.6	0.582
LVIDs (mm)	23.6 ± 6.0	26.6 ± 4.7	**<0.001**
IVSd (mm)	9.4 ± 2.2	8.4 ± 1.3	**<0.001**
PWd (mm)	8.1 ± 1.6	7.4 ± 1.2	**0.004**
RWT	0.36 ± 0.07	0.33 ± 0.05	**<0.001**
LAVi (ml/m^2^)	25.9 ± 9.3	25.1 ± 6.6	0.527
Transmitral E wave (cm/s)	103.8 ± 22.8	95.8 ± 17.0	**0.017**
Transmitral A wave (cm/s)	72.0 ± 23.5	61.5 ± 17.0	**0.002**
E/A	1.5 ± 0.4	1.7 ± 0.5	0.082
DT (ms)	171 ± 32	168 ± 36	0.561
Mitral lateral s’ (cm/s)	10.4 ± 2.4	11.4 ± 2.7	**0.024**
Mitral lateral e’ (cm/s)	15.6 ± 3.8	18.5 ± 3.7	**<0.001**
Mitral lateral a’ (cm/s)	8.0 ± 2.8	7.8 ± 2.8	0.680
E/e’	7.3 ± 4.1	5.3 ± 1.6	**<0.001**
RV basal diameter (mm)	28.5 ± 4.0	29.0 ± 3.6	0.453
TAPSE (mm)	23.9 ± 3.7	24.1 ± 3.1	0.698
RAVi (ml/m^2^)	22.5 ± 7.0	22.3 ± 6.6	0.867
PASP (mmHg)	25.6 ± 5.8	24.2 ± 4.7	0.226

Data are presented as mean ± SD. Values with a significant difference are presented in bold. LVIDd, left ventricular end-diastolic diameter; LVIDs, left ventricular end-systolic diameter; IVSd, interventricular septal thickness; PWd, posterior wall thickness; RWT, relative wall thickness; LAVi, left atrial volume index; DT, deceleration time; RV, right ventricular; TAPSE, tricuspid annular plane systolic excursion; RAVi, right atrial volume index; PASP, pulmonary arterial systolic pressure.

LV end-diastolic diameter (LVIDd) did not differ between the transplant and control groups, while LV end-systolic diameter (LVIDs) was significantly lower in the transplant patients. However, both the interventricular septal thickness (IVSd) and the posterior wall thickness (PWd) were shown to be higher in the KTX group and the RWT was also higher in them.

Both the transmitral E wave and A waves were significantly increased in the KTX patients, while the E/A ratio did not differ, and neither did the DT. The mitral lateral s’ and e’ were significantly lower in the transplanted group, on the other hand, the mitral lateral a’ did not show any difference. Accordingly, E/e’ was significantly higher in the KTX group.

Neither the RV basal diameter, the TAPSE nor the PASP showed any difference between the transplanted and the control patients.

Regarding atrial dimensions, LAVi and RAVi did not differ between the two groups.

### 3.3. 3D volumetric measures and LV speckle-tracking echocardiography

The 3D and LV speckle tracking data are shown in Table 3.

**Table 3:** Comparison of 3D and speckle-tracking echocardiographic data in the kidney transplant and the control groups.

	Kidney transplant (*n* = 74)	Control (*n* = 74)	*p*-value
3D LVEDVi (ml/m^2^)	67.39 ± 16.63	61.20 ± 8.92	**0.006**
3D LVESVi (ml/m^2^)	27.33 ± 8.73	23.63 ± 4.26	**0.002**
3D LVSVi (ml/m^2^)	39.55 ± 9.70	37.62 ± 6.46	0.159
3D LVEF (%)	60.02 ± 6.00	61.37 ± 3.69	0.106
3D LVMi (g/m^2^)	79.94 ± 16.58	66.17 ± 9.43	**<0.001**
3D LVGLS (%)	−20.52 ± 3.00	−21.95 ± 1.66	**<0.001**
3D LVGCS (%)	−29.74 ± 4.26	−28.61 ± 9.98	0.375
SDI (%)	7.76 ± 11.22	6.22 ± 10.02	**0.015**
3D LV Twist (°)	11.97 ± 7.32	9.33 ± 6.1	**0.019**
3D LV Torsion (°/cm)	1.45 ± 0.95	1.21 ± 0.70	0.079
3D RVEDVi (ml/m^2^)	68.35 ± 18.11	60.67 ± 10.52	**0.003**
3D RVESVi (ml/m^2^)	28.49 ± 9.52	24.02 ± 5.80	**0.001**
3D RVSVi (ml/m^2^)	38.25 ± 10.24	36.65 ± 6.73	0.269
3D RVEF (%)	58.79 ± 5.67	60.59 ± 4.34	**0.034**

Data are presented as mean ± SD. Values with a significant difference are presented in bold. LVEDVi, left ventricular end-diastolic volume index; LVESVi, left ventricular end-systolic volume index; LVSVi, left ventricular stroke volume index; LVEF, left ventricular ejection fraction; LVMi, left ventricular mass index; LVGLS, left ventricular global longitudinal strain; LVGCS, left ventricular global circumferential strain; SDI, systolic-dyssynchrony index; LV, left ventricular; RVEDVi, right ventricular end-diastolic volume index; RVESVi, right ventricular end-systolic volume index; RVSVi, right ventricular stroke volume index; RVEF, right ventricular ejection fraction.

**Figure 1:**
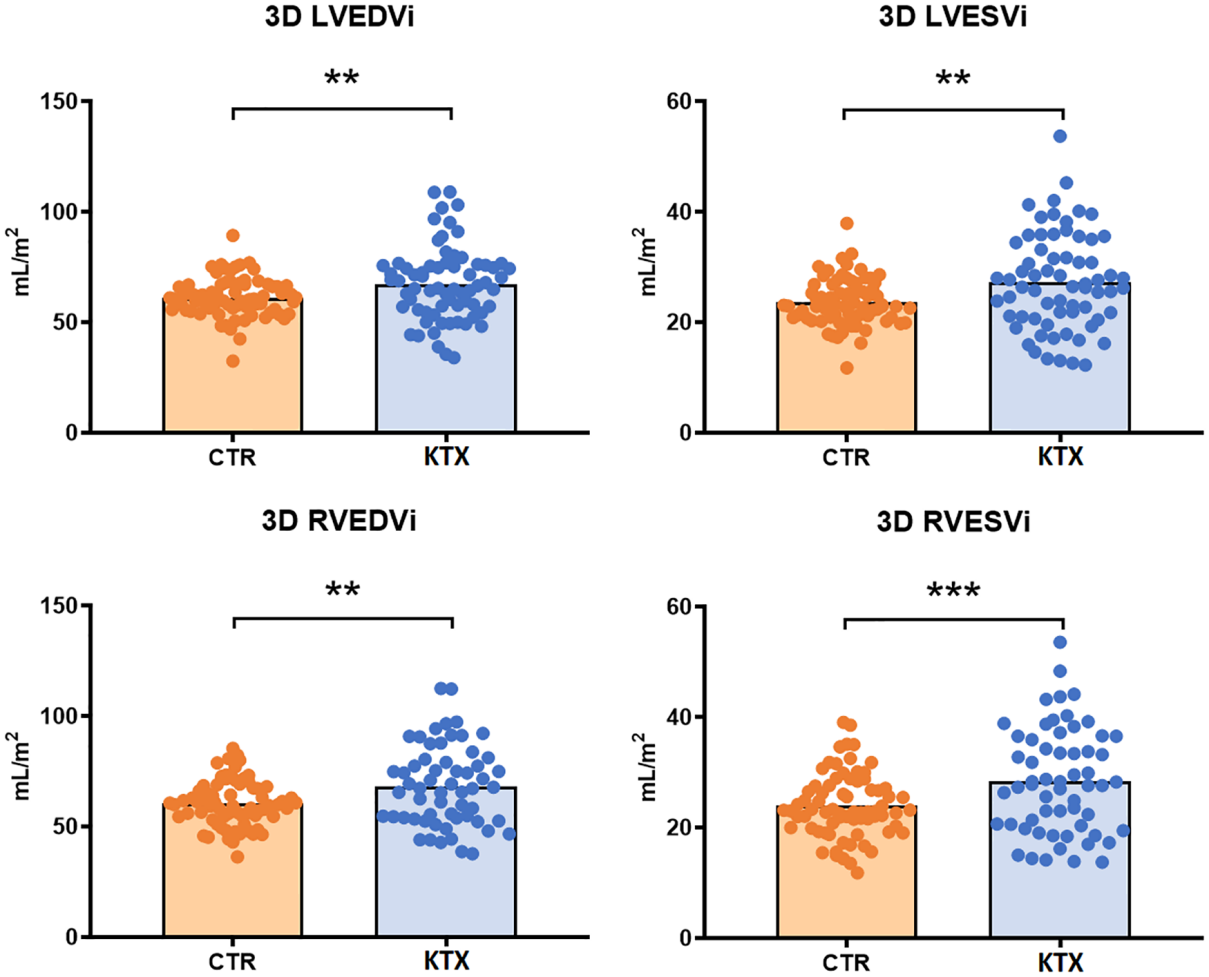
3D ventricular morphological measures of the two study groups. LVEDVi, LVESVi, RVEDVi and RVESVi were all significantly increased in the transplant group. LVEDVi, left ventricular end-diastolic volume index; LVESVi, left ventricular end-systolic volume index; RVEDVi, right ventricular end-diastolic volume index; RVESVi, right ventricular end-systolic volume index; CTR, control group; KTX, kidney transplant group; * = *p* < 0.05; ** = *p* < 0.01; *** = *p* < 0.001.

Both the LVEDVi and LVESVi were significantly increased in the transplant group (Figure 1). However, neither the LVSVi, nor the LVEF showed any statistically significant difference. Nevertheless, the LVMi was significantly increased in the KTX patients.

**Figure 2:**
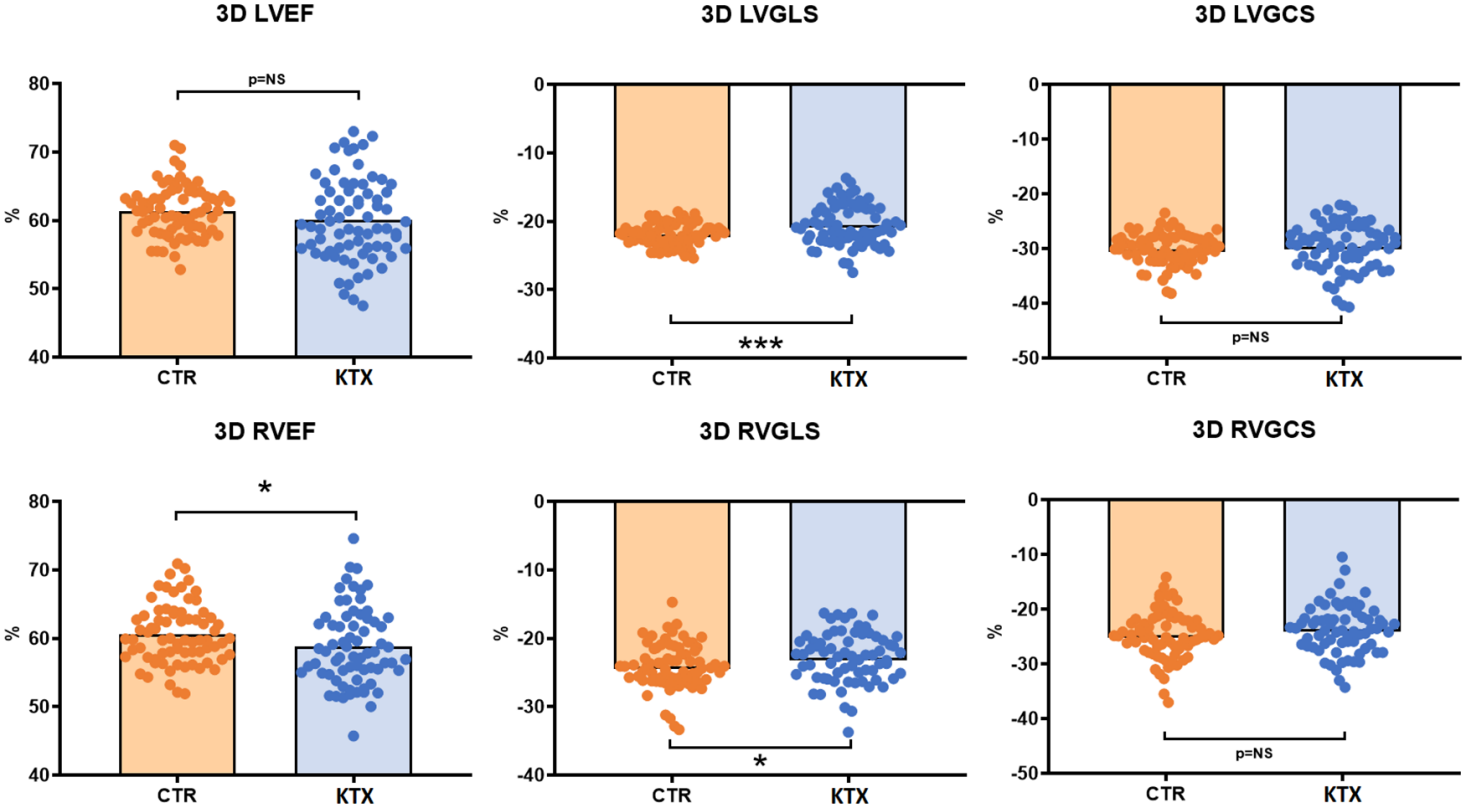
3D ventricular functional parameters of the two study groups. LVGLS was impaired in the transplant recipients, while LVEF and LVGCS did not differ. RVEF and RVGLS were significantly reduced in the transplant patients, while the RVGCS was comparable between the groups. LVEF, left ventricular ejection fraction; LVGLS, left ventricular global longitudinal strain; LVGCS, left ventricular global circumferential strain; RVEF, right ventricular ejection fraction; RVGLS, right ventricular global longitudinal strain; RVGCS, right ventricular global circumferential strain; CTR, control group; KTX, kidney transplant group; * = *p* < 0.05; ** = *p* < 0.01; *** = *p* < 0.001.

**Figure 3:**
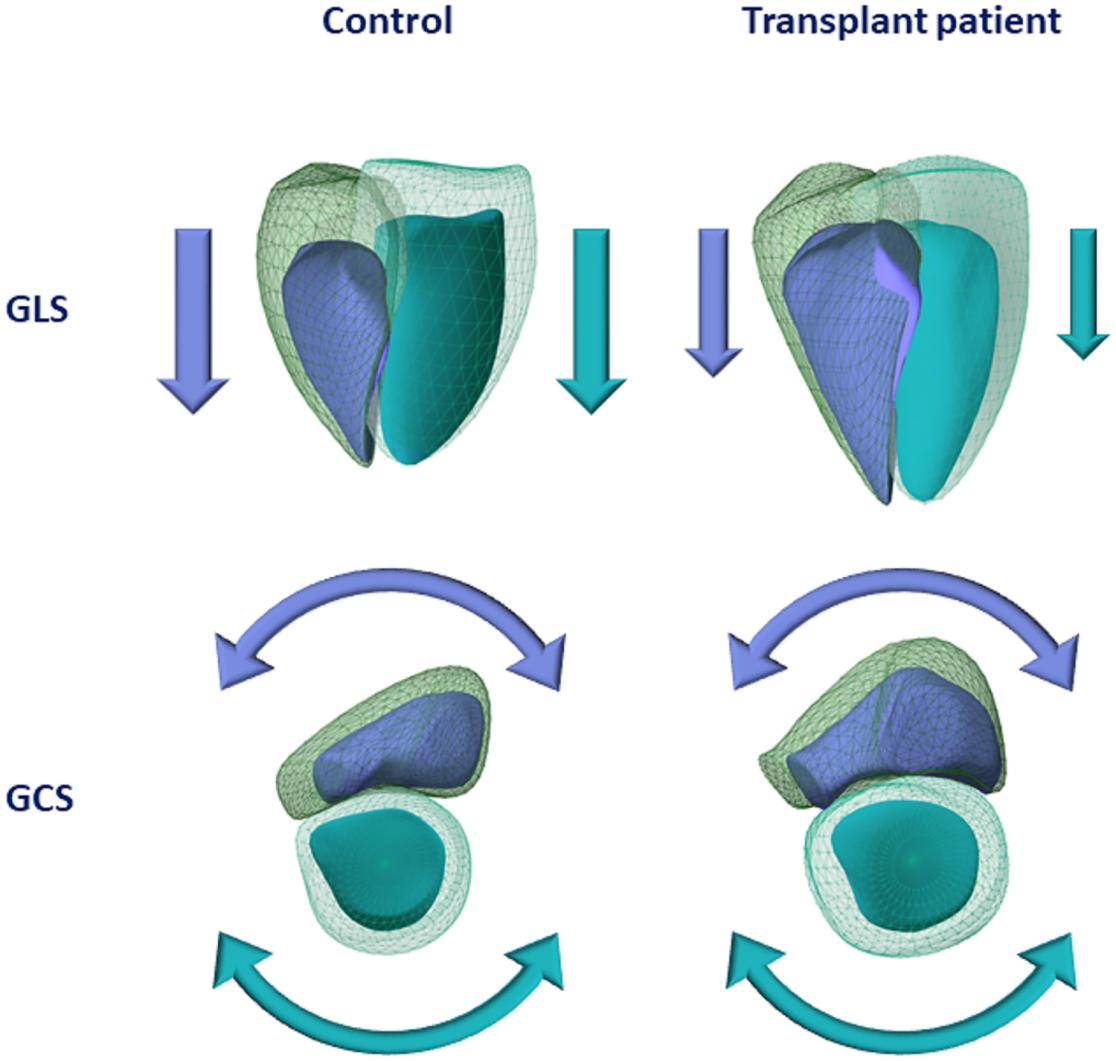
Graphical representation of a healthy control and a kidney transplant recipient in terms of three-dimensional left and right ventricular volumes and mechanics. The transplant patient shows larger left and right ventricular end-diastolic and end-systolic volumes compared with the healthy control (light turquoise mesh—left ventricular end-diastolic volume; dark turquoise surface—left ventricular end-systolic volume; green mesh—right ventricular end-diastolic volume; blue surface—right ventricular end-systolic volume). Concerning systolic function, both left and right ventricular global longitudinal strain values showed visible decrease in the transplant patient, whereas left and right ventricular global circumferential strain values were found to be comparable between the two subjects. GLS, global longitudinal strain; GCS, global circumferential strain.

LVGLS was decreased in the transplant patients, while the LVGCS did not differ (Figures 2, 3). SDI was significantly higher in the KTX patients, and so was the LV twist, however, the LV torsion did not show any difference.

RVEDVi and the RVESVi were both increased in the transplant group (Figure 1), although the RVSVi did not differ, whereas the RVEF was significantly decreased in the KTX group.

### 3.4. 3D RV deformation values

The data of the global and segmental 3D RV deformation measures are presented in Table 4.

**Table 4:** Comparison of 3D right ventricular data measured by our custom ReVISION method in the kidney transplant and the control groups.

	Kidney transplant (*n* = 74)	Control (*n* = 74)	*p*-value
3D RVGLS (%)	−22.81 ± 3.66	−24.11 ± 3.34	**0.030**
3D RVGCS (%)	−23.67 ± 4.49	−24.81 ± 4.38	0.129
3D RVGAS (%)	−39.85 ± 5.31	−42.20 ± 3.95	**0.003**
SCS (%)	−16.65 ± 5.31	−18.78 ± 5.34	**0.020**
SLS (%)	−21.42 ± 4.88	−22.99 ± 5.41	0.074
SAS (%)	−0.36 ± 0.07	−0.39 ± 0.07	**0.003**
FWCS (%)	−23.82 ± 4.48	−24.95 ± 4.39	0.137
FWLS (%)	−27.44 ± 4.81	−28.41 ± 5.05	0.249
FWAS (%)	−0.52 ± 0.06	−0.53 ± 0.06	0.197

Data are presented as mean ± SD. Values with a significant difference are presented in bold. RVGLS, right ventricular global longitudinal strain; RVGCS, right ventricular global circumferential strain; RVGAS, right ventricular global area strain; SCS, septal circumferential strain; SLS, septal longitudinal strain; SAS, septal area strain; FWCS, free wall circumferential strain; FWLS, free wall longitudinal strain; FWAS, free wall area strain.

3D RVGLS was significantly reduced in the KTX patients, while the RVGCS did not differ between the groups (Figures 2, 3). The RVGAS was lower in the transplanted patients.

The septal longitudinal strain (SLS) did not show any difference, however, the septal circumferential (SCS) and area (SAS) strains were significantly reduced in the KTX group. The free wall circumferential (FWCS), longitudinal (FWLS) and area strains (FWAS) did not differ between the groups.

### 3.5. The relationship between echocardiographic parameters and clinical data in the transplant patients

As shown in Table 5, the length of dialysis did not correlate with either 3D LVEF, 3D LVGLS, 3D LVGCS, 3D RVEF, or 3D RVGLS. However, it showed significant, albeit weak to moderate correlations with 3D RVGCS and various segmental 3D RV deformation measures, including SCS, SLS, SAS, FWCS, FWLS and FWAS.

**Table 5:** Correlations between 3D echocardiographic parameters and the length of dialysis.

	Versus length of dialysis	
	*r*-value	*p*-value
3D LVEF	−0.124	0.392
3D LVGLS	−0.024	0.868
3D LVGCS	−0.029	0.844
3D RVEF	−0.086	0.554
3D RVGLS	0.272	0.056
3D RVGCS	0.320	**0.023**
SCS	0.333	**0.018**
SLS	0.330	**0.019**
SAS	0.371	**0.008**
FWCS	0.331	**0.019**
FWLS	0.380	**0.006**
FWAS	0.371	**0.008**

Values with a significant correlation are presented in bold. LVEF, left ventricular ejection fraction; LVGLS, left ventricular global longitudinal strain; LVGCS, left ventricular global circumferential strain; RVEF, right ventricular ejection fraction; RVGLS, right ventricular global longitudinal strain; RVGCS, right ventricular global circumferential strain; SCS, septal circumferential strain; SLS, septal longitudinal strain; SAS, septal area strain; FWCS, free wall circumferential strain; FWLS, free wall longitudinal strain; FWAS, free wall area strain.

A handful of meaningful laboratory parameters also showed correlation with the 3D echocardiographic parameters. These findings are presented in Supplementary Table S2. The serum calcium levels showed significant relationship with 3D LV twist and torsion, while the serum phosphorus levels correlated significantly with 3D LVEDVi, LVESVi, LVSVi, LVMi and 3D RVSVi, also, the CaxP product correlated well with 3D LVEDVi and LVSVi. Furthermore, the serum magnesium levels showed direct relationship with 3D LVEDVi, LVESVi, LVSVi and LVMi, while also correlating with 3D RVEDVi and RVSVi. The creatinine levels showed significant, albeit weak, correlation with 3D LVESVi, LVMi and LVEF. On the other hand, GFR did not show any relationship with the 3D LV or RV morphological or functional parameters. The urinary protein to creatinine ratio correlated weakly, but statistically significantly with 3D LVESVi, LVMi, LVEF, LVGCS, RVGLS and RVGCS. We found no significant difference in terms of LV or RV morphological and functional measures between the hypertensive and non-hypertensive patients, as presented in Supplementary Table S3. Nevertheless, we are likely not powered to detect a difference by dividing the KTX group to subgroups. When comparing patients who receive steroids as part of the immunosuppressive regime, and patients who do not, our results showed that people on steroids had significantly larger 3D LVEDVi, LVESVi, LVMi, RVEDVi and RVESVi, however, there was no difference in the functional parameters, as shown in Supplementary Table S4.

### 3.6. Intra- and interobserver variability

The intra- and interreader variability analysis of our key 3D parameters demonstrated good agreement in the assessment of these measures (Supplementary Table S5).

## 4. Discussion

KTX is the treatment of choice in pediatric patients with ESKD, since it carries the best long-term prognosis in this population (2). Nevertheless, these patients have an increased risk of developing CVD due to multiple KTX-related risk factors, i.e., the cardiovascular effects of the immunosuppressive agents, progressively deteriorating graft function, hypertension and various acquired metabolic disorders, such as diabetes, hyperuricaemia and hyperlipidaemia (6–12, 20–22). Echocardiography is the mainstay modality to identify the possible cardiac involvement in this population, and state-of-the-art measurement methods, such as 3D echocardiography, may demonstrate a real additive value in this patient group as well (10–12, 16, 19, 23). According to our results, 3D assessment of the biventricular morphology and function can unveil subtle alterations of the pediatric KTX patients, even when the most commonly used conventional measures fail to demonstrate any difference. Moreover, these parameters also show a handful of correlations with clinical and laboratory characteristics of the population.

Our findings that the transplanted children grew up to be shorter compared to their age- and gender-matched peers, are in unison with evidence that CKD in childhood impairs growth (24, 25), and even after a successful KTX most recipients do not reach their calculated target height (26). The impairment in the physiological development during childhood may be an important factor in the alterations of cardiac morphology and function by itself (27).

In accordance with previous studies, a large proportion of our KTX patients had treated, albeit existing hypertension (28). Its clinical relevance lies in its association with decreased patient and graft survival after transplantation (29, 30). Interestingly however, we found that within the KTX group those who received antihypertensive therapy and those who did not, had comparable ventricular size and function. Adequate control of elevated blood pressure in this population is a major challenge (31), nevertheless, our findings support its importance due to the preserved LV and RV measures in our population.

In CKD and after KTX, LV hypertrophy is a common finding with conventional echocardiographic measures as well, and shows association with poor cardiovascular outcomes (23). Accordingly, we found increased IVSd and PWd in the transplant patients. RWT was also higher in the recipients, and so was 3D LVMi, which supports the presence of a concentric type of LV hypertrophy. While hypertensive KTX subjects had comparable LVMi to non-hypertensives, other factors also have to be taken into consideration: such as the hypertrophy-promoting effect of immunosuppressive agents, including calcineurin inhibitors and steroids (32, 33). Our findings supported this, as the steroid-receiving subgroup had significantly higher ventricular volumes and LVMi than those, who did not receive steroids. A major influence of hypertrophic remodeling may be the FGF-23 (fibroblast growth factor-23), a protein significantly involved in the cardio-renal axis in CKD (34). Previous studies showed that high serum levels of FGF-23 were associated with more advanced LV remodeling in ESKD patients (35).

LV hypertrophy is also commonly associated with diastolic dysfunction, which is also demonstrated in the KTX group by altered transmitral velocities and Tissue Doppler values (36–39).

While conventional, linear measures of LV and RV dimensions were comparable between the two study groups, 3D LV and RV volumes were significantly higher in the KTX group, suggesting dilation of the ventricles. The steroid-receiving KTX subgroup also presented with increased LV and RV dimensions, suggesting the contribution of immunosuppressive therapy to the observed findings (33). In the KTX patients LV structural abnormalities were not accompanied by changes in 3D LVEF. On the other hand, 3D speckle-tracking-derived LVGLS was significantly reduced, suggesting subclinical systolic dysfunction. Impaired LVGLS is associated with an increased risk of CV mortality both in CKD and KTX patients (40, 41). In parallel with our findings, in a study enrolling adult KTX patients, deterioration of LV systolic function persists even after successful KTX (42). Several factors may contribute to this: hypertension, impaired graft function and CKD-associated volume overload are all established factors of LVGLS impairment (43–45). Importantly, in adult patients reduced LVGLS is associated with cardiovascular events and mortality after KTX while LVEF is not, which underpins the necessity of LV deformation measurements in this population (40).

Regarding RV function, TAPSE did not differ between KTX and controls, however, 3D RVEF, RVGLS and RVGAS were impaired along with comparable 3D RVGCS. Previous 2D speckle-tracking echocardiography studies enrolling adult patients demonstrated improvement of RV longitudinal deformation following KTX (46). According to our results, 3D RVGLS remains impaired compared to a healthy cohort of subjects. As RVGCS and RVGAS can only be assessed by novel, custom 3D echocardiographic methods, data are scarce regarding these parameters. In a previous study by Kitano et al. (47), RVGCS and RVGAS were independent determinants of major cardiac adverse events. Nevertheless, the findings of an adult heart disease population can barely be extrapolated to our cohort.

Assessment of regional RV function may unveil further aspects of ventricular mechanics: in our study, transplant recipients had impaired 3D RV SCS and SAS, suggesting a more pronounced deterioration of septal movement components. Along with the impaired GLS in both ventricles, the septal predominance of this functional impairment indicate an origin of direct myocyte damage, rather than the effect of altered hemodynamic measures affecting the myocardium with the highest working load, i.e., the subendocardium and the interventricular septum. Interestingly, LV measures did not correlate with the length of pre-transplant dialysis, while RVGCS and regional strain measures showed a direct relationship with it—longer dialysis needs correlated with more impaired deformation. In a previous study enrolling adult ESKD patients, RVEF showed a relatively strong correlation with the duration of dialysis (48). Based on our findings, only RV deformation measures showed relationship with the length of pre-transplant dialysis, suggesting that KTX may be also beneficial in terms of preserving global RV function. Nevertheless, the different dialysis duration between the pediatric and adult populations may also explain why only subclinical changes of RV mechanics showed relationship with dialysis length in our cohort.

ESKD is complicated by mineral bone disease that is characterized by abnormalities in calcium and phosphorus metabolism, as well as dysregulation of the FGF-23 and PTH hormonal axis (49, 50). As previously mentioned, beyond pathological calcium and phosphorus homeostasis, FGF-23 also strongly contributes to adverse LV remodeling, which may explain the correlations between the calcium, phosphorus levels and the CaxP product, and the various 3D morphological and functional cardiac measures (34, 35).

We found that magnesium levels are also directly correlated with a number of 3D biventricular parameters. Magnesium plays a key role in modulating neuronal excitation, intracardiac conduction, and myocardial contraction by various ion transporters, including potassium and calcium channels. It also participates in regulating vascular tone, atherogenesis and thrombosis, vascular calcification, and proliferation and migration of endothelial and vascular smooth muscle cells. Accordingly, magnesium has a considerable influence on the pathogenesis of CVD. As the kidney is the major regulator of magnesium homeostasis, kidney disorders can potentially lead to both magnesium depletion and overload, thus increasing the risk of CVD (51). The serum creatinine levels and urinary protein to creatinine ratio showed weak correlation with various 3D LV and RV measures. These laboratory measures are established prognostic markers of the risk for cardiovascular disease and mortality (52–54) and naturally, are also robust markers of renal function of the KTX patients.

### 4.1. Limitations

Our study has a number of limitations that should be acknowledged for adequate interpretation. Firstly, this is a single-centre, retrospective study with a limited number of cases—further multicentre expansion of the population would strengthen our findings. Secondly, the cross-sectional study design did not allow the assessment of the prognostic power of the 3D parameters. The ReVISION Method is currently not a commercially available software, however, it was previously validated against gold-standard cardiac magnetic resonance imaging. Despite discussing the possible role of FGF-23 in the ventricular remodeling, we did not measure its serum levels in our population. Lastly, cystatin C was not measured in our cohort, therefore, we calculated GFR using the form of CKiD U25 formula which does not implement this laboratory measure.

### 4.2. Conclusion

Using three-dimensional speckle tracking echocardiography, we found distinct morphological and functional changes in both ventricles in pediatric kidney transplant patients. With the ReVISION Method we also measured the changes of right ventricular motion components, which were previously undescribed in this population. However, the pathophysiological background and clinical relevance of our RV strain findings are still unclear and require further research. Nevertheless, our data support the use of a comprehensive echocardiographic protocol applying advanced techniques in the care of kidney transplant patients.

## Data Availability

The raw data supporting the conclusions of this article will be made available by the authors, without undue reservation.
