# Supplementary material for: Get to the heart of pediatric kidney transplant recipients: Evaluation of left- and right ventricular mechanics by three-dimensional echocardiography
Source: Front Cardiovasc Med. 2023 Mar 17;10:1094765. doi: 10.3389/fcvm.2023.1094765 (PMC10063872; doi:10.3389/fcvm.2023.1094765)
Supplement: Supplementary file 1 [file Datasheet1.docx]

SUPPLEMENTARY MATERIALS

**Supplementary Table 1: Additional clinical characteristics of the kidney transplant group (n=74)**

|  |  |
| --- | --- |
| Creatinine (μmol/L) | 88 [75-113] |
| eGFR (mL/min/1.73m^2^) | 66.8 [55.3-84.3] |
| CKD stages, n (%) |  |
| I | 15 (20) |
| II | 35 (47) |
| III | 20 (27) |
| IV | 1 (1) |
| V | 3 (4) |
| BUN (mmol/L) | 6.9 [5.1-9.1] |
| Calcium (mmol/L) | 2.4 [2.3-2.4] |
| Phosphor (mmol/L) | 1.1 [1.0-1.4] |
| Ca x P (mmol^2^/L^2^) | 2.76 [1.75-5.04] |
| Magnesium (mmol/L) | 0.8 [0.7-0.8] |
| PTH (ng/mL) | 71 [54-118] |
| Urinary protein (mg/L) | 122 [48-294] |
| Nephrectomy, n (%) | 8 (11) |
| Repeat kidney transplantation, n (%) | 7 (9) |
| Cadaveric donation, n (%) | 60 (81) |
| Antihypertensive therapy, n (%) | 47 (64) |
| ACEi/ARB | 17 (23) |
| BB | 29 (39) |
| MRA | 0 (0) |
| CCB | 28 (38) |
| Diuretic | 3 (4) |
| Alpha-1 blocker | 5 (7) |
| Immunosuppression regimen, n (%) | 74 (100) |
| Tacrolimus | 63 (85) |
| Mycophenolate mofetil | 37 (50) |
| Mycophenolic acid | 22 (30) |
| Everolimus | 1 (1) |
| Azathioprine | 1 (1) |
| Cyclosporine | 7 (9) |
| Sirolimus | 4 (5) |
| CNI | 70 (95) |
| Steroid | 26 (35) |

Data are presented as mean±SD, median (interquartile range) or number of patients

*Abbreviations*: eGFR: glomerular filtration rate; BUN = blood urea nitrogen; Ca x P: calcium-phosphate product; PTH: parathyroid hormone; ACEi: angiotensin-convertase inhibitor; ARB: angiotensin receptor blocker; BB: beta blocker; MRA: mineralocorticoid receptor antagonist; CCB: calcium channel blocker; CNI: calcineurin inhibitor

**Supplementary Table 2: Significant correlations between the laboratory and 3D echocardiographic parameters of the KTX patients**

|  | Calcium | Phosphor | Ca x P | Magnesium | Creatinine | Urinary protein / creatinine |
| --- | --- | --- | --- | --- | --- | --- |
| 3D LVEDVi | r=-0.077  p=0.522 | r=0.304  **p=0.009** | r=0.282  **p=0.016** | r=0.398  **p=0.001** | r=0.200  p=0.092 | r=0.177  p=0.199 |
| 3D LVESVi | r=-0.071  p=0.556 | r=0.254  **p=0.032** | r=0.227  p=0.055 | r=0.365  **p=0.002** | r=0.280  **p=0.017** | r=0.314  **p=0.021** |
| 3D LVSVi | r=-0.070  p=0.559 | r=0.293  **p=0.013** | r=0.278  **p=0.018** | r=0.357  **p=0.003** | r=0.091  p=0.450 | r=-0.013  p=0.929 |
| 3D LVMi | r=-0.187  p=0.116 | r=0.276  **p=0.019** | r=0.228  p=0.055 | r=0.436  **p<0.001** | r=0.251  **p=0.033** | r=0.291  **p=0.033** |
| 3D LVEF | r=-0.037  p=0.758 | r=0.002  p=0.985 | r=0.007  p=0.952 | r=-0.122  p=0.321 | r=-0.253  **p=0.032** | r=-0.320  **p=0.018** |
| 3D LVGLS | r=0.024  p=0.839 | r=-0.130  p=0.276 | r=-0.136  p=0.255 | r=0.019  p=0.876 | r=0.087  p=0.470 | r=0.223  p=0.104 |
| 3D LVGCS | r=0.060  p=0.619 | r=-0.010  p=0.935 | r=-0.009  p=0.938 | r=0.117  p=0.343 | r=0.223  p=0.059 | r=0.293  **p=0.031** |
| 3D LV Twist | r=-0.300  **p=0.011** | r=0.051  p=0.670 | r=-0.009  p=0.941 | r=-0.045  p=0.714 | r=-0.049  p=0.685 | r=-0.209  p=0.130 |
| 3D LV Torsion | r=-0.312  **p=0.008** | r=0.071  p=0.552 | r=0.011  p=0.930 | r=-0.096  p=0.438 | r=-0.080  p=0.504 | r=-0.221  p=0.108 |
| 3D RVEDVi | r=-0.046  p=0.706 | r=0.219  p=0.071 | r=0.201  p=0.097 | r=0.283  **p=0.022** | r=0.161  p=0.186 | r=0.153  p=0.269 |
| 3D RVESVi | r=-0.002  p=0.987 | r=0.158  p=0.194 | r=0.151  p=0.216 | r=0.224  p=0.073 | r=0.193  p=0.112 | r=0.213  p=0.121 |
| 3D RVSVi | r=-0.087  p=0.479 | r=0.261  **p=0.031** | r=0.236  p=0.051 | r=0.313  **p=0.011** | r=0.144  p=0.239 | r=0.097  p=0.485 |
| 3D RVEF | r=-0.128  p=0.294 | r=0.100  p=0.416 | r=0.074  p=0.545 | r=-0.017  p=0.892 | r=-0.101  p=0.410 | r=-0.216  p=0.116 |
| 3D RVGLS | r=0.123  p=0.319 | r=-0.095  p=0.440 | r=-0.073  p=0.556 | r=0.002  p=0.988 | r=0.244  **p=0.045** | r=0.281  **p=0.040** |
| 3D RVGCS | r=0.120  p=0.332 | r=-0.083  p=0.503 | r=-0.065  p=0.600 | r=0.140  p=0.269 | r=0.163  p=0.184 | r=0.363  **p=0.007** |

Values with a significant correlation are presented in bold.

*Abbreviations:* Ca x P: calcium-phosphate product; LVEDVi = left ventricular end-diastolic volume index; LVESVi = left ventricular end-systolic volume index; LVSVi = left ventricular stroke volume index; LVMi = left ventricular mass index; LVEF = left ventricular ejection fraction; LVGLS = left ventricular global longitudinal strain; LVGCS = left ventricular global circumferential strain; LV = left ventricular; RVEDVi = right ventricular end-diastolic volume index; RVESVi = right ventricular end-systolic volume index; RVSVi = right ventricular stroke volume index; RVEF = right ventricular ejection fraction; RVGLS = right ventricular global longitudinal strain; RVGCS = right ventricular global circumferential strain

**Supplementary Table 3: Comparison of hypertensive and non-hypertensive pediatric kidney transplantation patients**

|  | Hypertensive  (n=47) | Non-hypertensive (n=27) | p-value |
| --- | --- | --- | --- |
| 3D LVEDVi (ml/m^2^) | 66.91±18.21 | 65.11±14.46 | 0.665 |
| 3D LVESVi (ml/m^2^) | 27.34±9.13 | 25.73±8.10 | 0.456 |
| 3D LVMi (g/m^2^) | 81.51±17.31 | 75.81±13.71 | 0.153 |
| 3D LVEF (%) | 59.53±5.18 | 60.91±7.29 | 0.351 |
| 3D LVGLS (%) | -20.22±3.05 | -21.08±2.88 | 0.242 |
| 3D LVGCS (%) | -29.50±3.67 | -30.17±5.21 | 0.524 |
| 3D RVEDVi (ml/m^2^) | 67.76±20.21 | 61.76±15.18 | 0.195 |
| 3D RVESVi (ml/m^2^) | 28.53±9.87 | 25.39±8.75 | 0.184 |
| 3D RVEF (%) | 58.22±4.89 | 59.75±6.80 | 0.280 |
| 3D RVGLS (%) | -22.51±3.32 | -23.36±4.22 | 0.356 |
| 3D RVGCS (%) | -23.26±4.31 | -24.38±4.78 | 0.322 |

Data are presented as mean±SD

Values with a significant correlation are presented in bold.

*Abbreviations*: ; LVEDVi = left ventricular end-diastolic volume index; LVESVi = left ventricular end-systolic volume index; LVMi = left ventricular mass index; LVEF = left ventricular ejection fraction; LVGLS = left ventricular global longitudinal strain; LVGCS = left ventricular global circumferential strain; RVEDVi = right ventricular end-diastolic volume index; RVESVi = right ventricular end-systolic volume index; RVEF = right ventricular ejection fraction; RVGLS = right ventricular global longitudinal strain; RVGCS = right ventricular global circumferential strain

**Supplementary Table 4: Comparison of those pediatric kidney transplantation patients who receive steroids as immunosuppressant and those, who do not**

|  | Steroid (n=26) | No steroid (n=48) | p-value |
| --- | --- | --- | --- |
| 3D LVEDVi (ml/m^2^) | 73.84±16.17 | 61.06±14.49 | **0.001** |
| 3D LVESVi (ml/m^2^) | 29.87±9.33 | 24.69±7.68 | **0.013** |
| 3D LVMi (g/m^2^) | 87.39±17.77 | 74.24±12.48 | **0.038** |
| 3D LVEF (%) | 60.07±6.40 | 59.97±5.90 | 0.947 |
| 3D LVGLS (%) | -20.57±3.14 | -20.45±2.97 | 0.871 |
| 3D LVGCS (%) | -29.95±4.72 | -29.63±4.07 | 0.763 |
| 3D RVEDVi (ml/m^2^) | 73.40±20.13 | 60.35±15.95 | **0.004** |
| 3D RVESVi (ml/m^2^) | 30.31±9.87 | 25.58±8.25 | **0.047** |
| 3D RVEF (%) | 59.56±5.92 | 58.12±5.43 | 0.305 |
| 3D RVGLS (%) | -23.30±3.80 | -22.38±3.53 | 0.313 |
| 3D RVGCS (%) | -23.98±4.19 | -23.48±4.76 | 0.663 |

Data are presented as mean±SD

Values with a significant correlation are presented in bold.

*Abbreviations*: ; LVEDVi = left ventricular end-diastolic volume index; LVESVi = left ventricular end-systolic volume index; LVMi = left ventricular mass index; LVEF = left ventricular ejection fraction; LVGLS = left ventricular global longitudinal strain; LVGCS = left ventricular global circumferential strain; RVEDVi = right ventricular end-diastolic volume index; RVESVi = right ventricular end-systolic volume index; RVEF = right ventricular ejection fraction; RVGLS = right ventricular global longitudinal strain; RVGCS = right ventricular global circumferential strain

**Supplementary Table 5: Intra- and interobserver variability of the key parameters**

|  | **Intraobserver variability** | | **Interobserver variability** | |
| --- | --- | --- | --- | --- |
|  | **ICC** | **CV** | **ICC** | **CV** |
| **LVEDV** | 0.977 | 4.499 | 0.972 | 4.037 |
| **LVEF** | 0.920 | 3.108 | 0.823 | 5.739 |
| **LVGLS** | 0.961 | 2.973 | 0.838 | 7.450 |
| **LVGCS** | 0.759 | 8.756 | 0.799 | 8.927 |
| **RVEDV** | 0.979 | 4.425 | 0.917 | 10.277 |
| **RVEF** | 0.834 | 4.791 | 0.847 | 7.566 |

*Abbreviations*: ICC = intraclass correlation coefficient; CV = coefficient of variation; LVEDV = left ventricular end-diastolic volume; LVEF = left ventricular ejection fraction; LVGLS = left ventricular global longitudinal strain; LVGCS = left ventricular circumferential strain; RVEDV = right ventricular end-diastolic volume; RVEF = right ventricular ejection fraction
